# Nevirapine-Associated Early Hepatotoxicity: Incidence, Risk Factors, and Associated Mortality in a Primary Care ART Programme in South Africa

**DOI:** 10.1371/journal.pone.0009183

**Published:** 2010-02-17

**Authors:** Kathryn M. Chu, Andrew M. Boulle, Nathan Ford, Eric Goemaere, Valerie Asselman, Gilles Van Cutsem

**Affiliations:** 1 South African Medical Unit, Médecins Sans Frontières, Johannesburg, South Africa; 2 Centre for Infectious Disease Epidemiology and Research, School of Public Health and Family Medicine, Faculty of Health Sciences, University of Cape Town, Cape Town, South Africa; 3 Médecins Sans Frontières, Khayelitsha, Cape Town, South Africa; 4 Infectious Diseases Clinic, JF Jooste Hospital, Provincial Government of the Western Cape, Cape Town, South Africa; BMSI-A*STAR, Singapore

## Abstract

**Background:**

The majority of antiretroviral treatment programmes in sub-Saharan Africa are scaling up antiretroviral treatment using a fixed dose first-line antiretroviral regimen containing stavudine, lamivudine, and nevirapine. One of the primary concerns with the use of this regimen is nevirapine-associated hepatotoxicity.

**Methodology/Principal Findings:**

Study participants were 1809 HIV-infected, antiretroviral naïve adults initiating nevirapine-based antiretroviral therapy between November 2002 and December 2006. The primary outcome was early hepatotoxicity. Secondary outcomes were associations with hepatotoxicity and mortality at six months. The cumulative proportion of early hepatotoxicity ranged from 1.0–2.0% giving an incidence-rate at 102 days of 3.6–7.6 per 100 person-years. Median time to hepatotoxicity was 32 (IQR 28–58) days. At 12 weeks, only 8% of patients had alanine aminotransferase monitoring at all the time-points recommended by national guidelines. No association was found between age, gender, baseline CD4 count, concurrent tuberculosis infection, prior participation in a prevention of mother-to-child-transmission program, or baseline weight and early hepatotoxicity. There was no association between early hepatotoxicity and mortality.

**Conclusions:**

The cumulative proportion of early hepatotoxicity in nevirapine based antiretroviral therapy was low in this resource-constrained setting. Hepatotoxicity was not associated with mortality. Frequent routine monitoring of alanine aminotransferase proved difficult to implement in this public sector primary care programme. Focused monitoring in the first month may be a more cost-effective and pragmatic option in settings with limited resources. Correlation with clinical signs and symptoms may allow future alanine aminotransferase testing to be dictated by clinical criteria.

## Introduction

The majority of antiretroviral treatment programmes in sub-Saharan Africa are scaling up antiretroviral treatment (ART) using a fixed dose first-line antiretroviral regimens containing stavudine, lamivudine and nevirapine (NVP) [1], [2]. One of the primary concerns with the use of this regimen is nevirapine-associated hepatotoxicity (HT) [3], [4], [5], [6], [7], [8], [9], [10]. In resource rich countries, screening for HT during ART has been primarily based on serum level of alanine aminotransferase (ALT), a liver enzyme which serves as a “proxy” for liver inflammation and damage. Clinical guidelines recommend discontinuation of NVP at grade 3 or 4 HT [11]. ALT testing requires laboratory technology that is sophisticated and expensive and therefore not routinely available at the health-centre level in most resource-poor countries. In order to encourage a simplified approach to scaling up antiretroviral treatment, WHO Guidelines do not insist on ALT monitoring as a pre-requisite to ART provision at the primary care level, and recommendations for frequency of testing when it is available are unclear [1]. This, however, is in contrast to manufacturer guidelines that recommend vigilant ALT monitoring for NVP users for the first 18 weeks, and regular monitoring thereafter whilst on treatment [3]. Clinical trials support routine monitoring in the first 12 weeks [6], [12], [13], but it is unknown if routine ALT testing prevents morbidity or mortality in routine programme settings.

Alcohol and hepatitis C co-infection are well established risk factors for hepatotoxicity but the effects of other host characteristics are not well studied [14], [15]. While early clinical trials conducted in resource rich-settings found that female gender and baseline CD4 cell counts above 250 cells/µl are risk factors for hepatotoxicity, results from other studies are conflicting [6], [16], [17], [18].

In clinical trials, mostly conducted in resource-rich countries, NVP- associated HT ranges from 8–18% [7], [9], [14]. NVP- associated HT in routine programme settings in Africa appears to be lower (1–2%) [19], [20], [21], although these studies may under-represent the risk of HT because of limited ALT monitoring. The objectives of this study were to describe the incidence, risk factors, and associated mortality due to hepatotoxicity in a primary care ART programme in South Africa with frequent ALT monitoring.

## Methods

### Study Setting

This study included patients from three primary care HIV clinics in Khayelitsha township, South Africa, where antiretroviral treatment (ART) has been provided by Médecins Sans Frontières (MSF) since 2001 in collaboration with the Provincial Department of Health. These clinics follow national South African treatment guidelines which comply with WHO guidelines: all patients were started on either NVP or EFV-based ART. NVP-based regimens were initiated with the recommended lead-in dose of NVP 200 mg daily for 14 days followed by NVP 200 mg twice daily. ALT testing was offered to patients starting NVP-based ART at baseline, 2 weeks, 4 weeks, 8 weeks, 12 weeks, 24 weeks then every 6 months thereafter. Specimens for laboratory testing were transported the same day to a single regional laboratory run by the National Health Laboratory Services which applied stringent quality control measures. ALT assays were conducted using a spectrophotometer following standard operating procedures.

### Inclusion and Exclusion Criteria

All patients initiating NVP-based ART from November 2002 (when ALT testing became available) until the end of December 2006 and who were on NVP for at least four weeks were considered for analysis. For the analysis of HT, patients were stratified into two groups: those who had ALT monitoring for at least 4 weeks at all time points recommended by national guidelines (complete monitoring) and those with incomplete monitoring. Those who were treatment-experienced and children under 16 years old were excluded. Women who had previously received single or dual NVP-based therapy via the prevention of mother to child transmission (PMTCT) program were also included.

### Definitions

Serum ALT was graded according to criteria established by the AIDS Clinical Trial Group [22]: grade 0: <1.25x upper limit of normal (ULN); grade 1: 1.25–2.5x ULN; grade 2: 2.6–5.0x ULN, grade 3: 5.1–10x ULN; grade 4: >10x ULN. Baseline ALT was defined as any measurement between 90 days prior to 6 days after ART initiation; four week ALT was any measurement between 7 and 41 days after ART initiation; 8 week ALT between 42 and 69 days after ART initiation, and 12 week ALT between 70 and 102 days after ART initiation. Since few patients had ALT measurements at 2 weeks, these were combined with the 4 week measurements. If more than one ALT was measured during the visit time, the highest ALT was used. Early hepatotoxicity was defined as an increase in ALT from grade 0–2 at baseline to grade 3 or 4 within 102 days of starting ART.

### Time on NVP and Mortality

Patient time on NVP was calculated from treatment commencement to HT or until the drug was stopped or substituted for other reasons. Mortality was evaluated up to 6 months after initiation of NVP-based ART.

### Statistical Analysis

Data was exported from a database prospectively maintained for routine monitoring and evaluation. Serum ALT values were obtained from the laboratory and merged using Stata (Version 10, College Station, TX, USA). Baseline characteristics were described using medians and interquartile ranges (IQRs) for continuous variables and counts and percentages for categorical data. Means were compared using the student's t-test and proportions using the chi squared test. Person-time of observation were accrued from the initiation on NVP-based ART until either occurrence of death, loss to follow-up, transfer to another ART programme or censoring of observation in July 2007. Kaplan–Meier analysis was used to estimate cumulative HT. Cox proportional hazards were used to determine associations between baseline characteristics and HT and mortality. Logistic regression was used to determine associations between baseline characteristics and grade 3 or 4 ALT baseline elevation. Variables considered in the analysis for early HT and baseline ALT elevation included age, gender, baseline CD4 cell count (cells/µl), increase in CD4 count >100 cells/µl at 6 months, baseline weight less than 60 kg, concurrent TB treatment, and prior prevention of mother-to-child transmission (PMTCT) program enrolment. Early HT was also considered in the analysis for mortality. All tests and confidence intervals (CI) were considered to be significant at a p≤0.05 (two-sided). An additional chart review was conducted on patients with early HT to obtain information on viral hepatitis testing, associated symptoms, outcome of HT, drug regimen changes, and death attributable to HT.

### Ethics

Procedures for selection and inclusion of patients within the ARV program followed the national guidelines for the implementation and roll-out of ART. General measures were provided to ensure patient confidentiality, consent for HIV testing, and counselling and support for those who receive a positive HIV test results. ART and all laboratory testing services (including ALT) were offered free of charge. The University of Cape Town ethics committee gave approval for this study without requiring patient consent because the analysis was based on routine clinical data. No additional data were collected specifically for this study, and the data were fully de-identified prior to analysis.

## Results

### Patient Characteristics

From November 2002 to December 2006, 3728 HIV-infected, ART-naïve individuals were initiated on ART. Of these, 1831 (49%) were started on NVP- based first-line therapy; 1809 (99%) remained on NVP at 4 weeks. Of these 911 (50%) patients had at least a baseline and a 4 week ALT measurement (complete monitoring) and 898 (50%) had either a missing baseline or 4 week ALT measurement (incomplete monitoring). Patient characteristics of both these groups on NVP are shown in Table 1. The median baseline CD4 count was 112 (IQR 57–164) cells/µl. In the group with complete ALT monitoring, there were 216 (24%) males compared to 167 (19%) in the group with incomplete monitoring (p = 0.008), and the median follow-up time on NVP-based ART was 18 (IQR 8–26) months compared to 9 (IQR 5–19) months respectively (p<0.001).

**Table 1:** Characteristics of patients on nevirapine-based antiretroviral treatment*.

Characteristics	Total Cohort	Complete ALT Monitoring**	Incomplete ALT Monitoring***	p
Total on NVP at 4 weeks	1809	911 (50)	898 (50)	
Males	383 (21)	216 (24)	167 (19)	0.008
Age on starting NVP based ART, years	32 (28–37)	32 (28–38)	31 (27–37)	0.989
Baseline CD4+ count, cell/µl	112 (57–164)	117 (63–166)	107 (49–163)	0.972
Baseline viral load, log_10_ copies	5.0 (4.5–5.5)	5.0 (4.4–5.5)	5.0 (4.5–5.5)	0.161
WHO clinical staging on starting ART				
Stage 1	169 (9)	76 (8)	84 (9)	0.449
Stage 2	194 (11)	91 (10)	103 (11)	0.309
Stage 3	922 (51)	467 (51)	455 (51)	0.800
Stage 4	533 (29)	277 (30)	256 (28)	0.376
Weight on starting ART, kg	60 (53–69)	60 (53–69)	60 (53–69)	0.437
Follow-up time on NVP-based ART, months	13 (6–22)	18 (8–26)	9 (5–19)	<0.001
6 month mortality	73 (4)	16 (2)	57 (6)	<0.001

Continuous variables are given as medians (interquartile range). Ordinal and discrete variables are given as n(%). NVP, Nevirapine. ART, antiretroviral therapy. WHO, World Health Organization.

*On NVP for at least 4 weeks.

**Patients with at least baseline and 4 week ALT.

***Patients without baseline or 4 week ALT.

### ALT Grades and Baseline ALT Elevation

Of the 1831 patients on NVP at baseline, 1444 (79%) patients had a baseline ALT measurement. Of the 1809 patients on NVP at 4 weeks, 911(50%) had ALT measured at baseline and 4 weeks. Of the 1705 patients on NVP at 8 weeks, 368 (22%) had complete ALT monitoring (baseline, 4 and 8 weeks), and at 12 weeks, only 127 (8%) had all recommended ALT monitoring (baseline, 4, 8, and 12 weeks). ALT grades for these visits (0, 4, 8, and 12 weeks) are shown in Table 2. Eighty-eight percent of ALT measurements were not elevated (grade 0). Eight (0.6%) of 1444 patients had a baseline grade 3 or 4 ALT elevation. Of those with grade 3 or 4 baseline transaminitis, 4 (50%) continued NVP-based ART. Of these, all (100%) resolved their ALT elevations after 4 weeks. Baseline CD4 cell count <50 cells/µl was the only variable associated with baseline grade 3 or 4 transaminitis (unadjusted OR = 5.99, 95% CI 1.42–25.1) (data not shown).

**Table 2:** Alanine aminotransferase for patients with complete monitoring.

	Baseline	(%)	4 weeks	(%)	8 weeks	(%)	12 weeks	(%)
On NVP	1831		1809		1705		1665	
Complete ALT monitoring*	1444	(79)	911	(50)	368	(22)	127	(8)
ALT Grade 0	1314	(90)	779	(86)	321	(87)	105	(83)
ALT Grade 1	110	(8)	86	(9)	33	(9)	17	(13)
ALT Grade 2	13	(1)	32	(4)	9	(2)	3	(2)
ALT Grade 3	6	(0.4	5	(0.6)	3	(0.8)	2	(2)
ALT Grade 4	2	(0.1)	9	(0.1)	2	(0.5)	0	(0)

*Patients who had ALT monitoring according to South African guidelines up to the time of consultation. The guidelines recommend ALT at 0, 4, 8, and 12 weeks.

All variables are given as n(%). NVP, nevirapine. ALT, alanine liver transferase. ALT grade 0:<1.25x upper limit of normal (ULN); grade 1: 1.25–2.5x ULN; grade 2: 2.6–5.0x ULN, grade 3: 5.1–10x ULN; grade 4: >10x ULN.

### Hepatotoxicity

**Figure 1:**
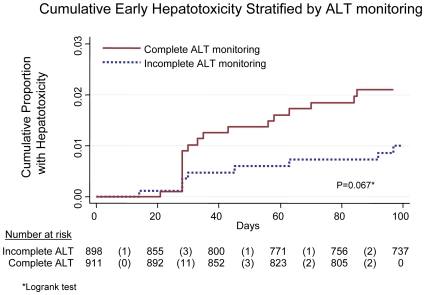
Cumulative early hepatotoxicity stratified by ALT monitoring.

Twenty-six patients developed early HT. The median time to early HT was 32 (IQR 28–63) days. Among patients who had complete ALT monitoring, the cumulative proportion of early HT was 2.0% by 102 days on NVP giving an incidence rate of 7.6 (95% CI: 4.8–12.1) per 100 person-years over this period. The cumulative proportion with HT in patients with incomplete ALT monitoring was 1.0% by 102 days on NVP giving an incidence-rate of 3.6 (95% CI: 1.8–7.2) per 100 person-years over this period (Figure 1).

Of the 26 patients with early HT, 14 (54%) had grade 3 HT and 12 (46%) had grade 4 HT. Four (15%) were being treated for active tuberculosis. Eight (31%) inadvertently continued NVP-based ART of which 7 (88%) resolved their HT and one was LTFU. Of the 18 who stopped NVP, all switched to efavirenz (EFV) based-ART. Of these, 2 (12%) developed further HT on EFV. Symptoms were known for 17 (65%). Of these 8 (47%) had either a rash or symptoms consistent with hepatitis (Table 3). Eleven additional patients developed late hepatotoxcity (after 12 weeks) between 121–891 days.

**Table 3:** Patients with early hepatotoxicity on nevirapine-based antiretroviral treatment.

Patient Number	Age	Gender	Baseline CD4	6 month CD4	Grade HT	ALT Monitoring	Tuberculosis co-infection	Symptoms	Viral Hepatitis co-infection	NNRTI after HT diagnosis	Outcome of HT	6 month patient outcome
1	50	Female	100		4	Complete	No	Rash	Not excluded	EFV	Resolved	Dead, HT
2	26	Female	189	605	4	Complete	No	No	Excluded	EFV	Resolved	Alive
3	32	Female	4		3	Complete	No	No	Not excluded	NVP	Resolved	Alive
4	29	Female	219	358	3	Complete	No	Unknown	Not tested	NVP	Resolved	Alive
5	28	Female	191		4	Complete	No	Rash and hepatitis	Excluded	EFV	Resolved	LTFU
6	30	Female	190	320	4	Complete	No	Unknown	Not tested	EFV	Resolved	Alive
7	28	Female	172	266	4	Complete	No	Hepatitis	Excluded	EFV	Resolved	Alive
8	32	Female	178	682	4	Complete	No	No	Not excluded	EFV	Resolved	Alive
9	48	Female	79		4	Complete	Yes	No	Not excluded	EFV	Resolved	Dead, not HT
10	35	Male	100	210	4	Complete	No	Rash	Not excluded	EFV	Resolved	Alive
11	38	Male	154	283	3	Complete	No	No	Not excluded	EFV	Resolved	Alive
12	25	Female	92	245	3	Complete	No	No	Excluded	EFV	Continued	Alive
13	48	Female	47	74	3	Complete	No	Unknown	Not tested	EFV	Resolved	Alive
14	48	Male	5	116	3	Complete	No	Unknown	Not tested	EFV	Resolved	Alive
15	23	Male	29	89	3	Complete	Yes	Unknown	Not tested	NVP	Resolved	Alive
16	41	Female	190	405	4	Complete	No	No	Not excluded	NVP	Resolved	Alive
17	28	Female	60		4	Complete	No	Unknown	Not tested	EFV	Resolved	Alive
18	32	Female	36	413	3	Complete	No	Rash	Not excluded	NVP	Resolved	Alive
19	34	Female	66		4	Incomplete	Yes	Rash	Not excluded	EFV	Resolved	Dead, not HT
20	36	Female	134	271	3	Incomplete	No	Rash	Not excluded	NVP	Resolved	Alive
21	31	Male	180	423	3	Incomplete	No	Unknown	Not tested	EFV	Resolved	Alive
22	30	Male	2		4	Incomplete	No	Unknown	Not tested	EFV	Continued	Alive
23	38	Female	78		3	Incomplete	No	No	Not excluded	EFV	Resolved	Alive
24	37	Female	41	179	3	Incomplete	Yes	No	Not excluded	NVP	Resolved	Alive
25	36	Female	217		3	Incomplete	No	Rash	Excluded	EFV	Resolved	Alive
26	33	Female	148	286	3	Incomplete	No	Unknown	Not tested	NVP	Resolved	Alive

HT, Hepatotoxicity. ALT, alanine aminotransferase. NNRTI, non-nucleoside reverse transcriptase inhibitor. EFV, efavirenz. NVP, nevirapine.

### Risk Factors for Hepatotoxicity

There were no associations found between baseline characteristics and hepatotoxicity including gender (adjusted hazards ratio, AHR = 1.2, P = 0.756), age (AHR = 1.0, P = 0.540), baseline CD4 count (AHR = 1.0, P = 0.834), and increase in CD4 count >100 cells/µl at 6 months (AHR = 0.9, P = 0.489). (Table S1).

### Mortality

Seventy-three (4%) of 1809 patients died within 6 months of starting NVP based ART. In patients with at least baseline and 4 week ALT measurements (complete monitoring), 16/911 (2%) died in the first 6 months. In this group, 18/911 patients had early HT, of whom 2/18 (11%) died. One of these deaths was HT-related. In the patients with incomplete ALT monitoring, 57/898 (6%) died in the first 6 months. In this group, 8/898 had early HT, of whom one (12%) died, of non-HT causes. All three patients who died with early HT had grade 4 ALT elevations. There was no mortality at 6 months in patients who developed late HT.

Baseline CD4 count >100 cells/µl was protective against mortality (AHR = 0.44, P<0.001) on multivariate analysis. Variables not associated with mortality included age (AHR = 1.3, P = 0.412), gender (AHR = 1.0, P = 0.345), HT (AHR = 2.5, P = 0.126), ALT elevation at baseline (AHR = 2.4, P = 0.383), current TB status (unadjusted HR = 0.9, P = 0.807), and PMTCT attendance (unadjusted HR = 0.9, P = 0.184). (Table S2).

## Discussion

The cumulative proportion of early HT was 1.0–2.0% and not associated with increased mortality. This is comparable to low incidence proportions reported by Hahn et al (2.2%) from an ARV program in Uganda and Meyssonnier et al (1.2%) in a program from Niger [18], [20]. In contrast, clinical trials with serial ALT testing have reported higher HT. For example, in the Boehringer Ingelheim trial, of 2545 patients on NVP, 10% developed HT at one year [7] and in a South African trial 17% developed HT within 3 months [6].

Programmatic ALT monitoring was enforced in less than one tenth of patients at 3 months. This highlights the difficulty of implementing serial ALT monitoring in high-burden public sector clinics. Even when monitoring schedules were adhered to, the proportion of patients with severe hepatotoxicity was very low. Furthermore, patients with ALT elevations who were also symptomatic could have been identified by their symptoms. Finally, many patients who had HT by ALT elevation were left on NVP due to administrative delays in receiving the laboratory results and subsequently revert to normal levels. This study did not demonstrate that ALT monitoring according to national guidelines adverted severe or fatal hepatotoxicity. The one HT-related death occurred in a patient with complete monitoring.

The medium time to early HT was 32 days and was consistent with other studies [6], [23]. Time to late HT (after 12 weeks) was highly variable and did not account for any additional mortality. This suggests programmatic ALT monitoring is most valuable in the first month and continued testing after 12 weeks can be of lower priority in resource constrained settings.

We did not identify any risk factors for HT. In particular, a higher CD4 was not associated with HT. This contrasts with previous findings that men and women with CD4 counts greater than 400 cells/µl and 250 cells/µl, respectively, are at greatest risk [24]. In sub-Saharan Africa, especially in the programmatic setting, baseline CD4 counts are generally much lower than in resource-rich countries because of difficulties in accessing care [25]. Because our median CD4 count was low (112 cells/µl [IQR 57–164]) we may not have been able to demonstrate this association. However, recent studies with higher median CD4 counts suggest that CD4 count is not associated with HT [17], [18]. Patient gender was not a risk factor for HT. This agrees with results from other programmatic settings [16], [23] but conflicts with clinical trials [6].

This study had a number of strengths and limitations. First, this is one of the largest cohorts of patients on NVP-based ART in southern Africa. Second, laboratory ALT values were used to determine HT so misclassification was unlikely. Finally, the availability of a group with complete ALT monitoring allowed calculation of ALT grades by weeks on NVP. The main limitation was that there were many patients with incomplete ALT monitoring and transient HT may have been missed which likely accounted for the lower incidence rate of early HT in this group. However, the incidence rate in the group with complete monitoring likely reflects true early HT. Another limitation was that other risk factors for HT such as alcohol use and hepatitis co-infection could not be evaluated [5], [7], and this may have confounded the overall incidence estimate: rates of hepatitis co-infection reported in South Africa [26] are lower than settings where hepatitis co-infection has been found to be a risk factor [5]. Low BMI has been reported as a risk factor [6]; this could not be assessed in our study as height was not routinely recorded. Finally, data on clinical manifestations of NVP hypersensitivity were incomplete.

We believe our findings have important public health implications regarding ALT monitoring and the risk of nevirapine-based ART regimens. The risk of life-threatening hepatotoxicity has led NVP manufacturers to warn against its use without close serological and clinical monitoring and in the US and Europe serial serum ALT testing is routine. In resource-limited settings such as in sub-Saharan Africa where NVP is the mostly commonly used NNRTI, ALT monitoring is expensive and often technologically not feasible and its use in routine programmes needs to be carefully considered. In our cohort in South Africa, the incidence rate of early HT is low in this population with a low median baseline CD4 count. Routine ALT monitoring was not consistently implemented and not associated with mortality. Taking into account these considerations, focused monitoring in the first month may be a more cost-effective and pragmatic option in settings with limited resources. Correlation with clinical signs and symptoms may allow future ALT testing to be dictated by clinical criteria.

## Supporting Information

Table S1 Associations between patient characteristics and hepatotoxicity.
(0.06 MB DOC)

Table S2 Associations between patient characteristics and mortality.
(0.06 MB DOC)
