# Supplementary material for: Nevirapine-Associated Early Hepatotoxicity: Incidence, Risk Factors, and Associated Mortality in a Primary Care ART Programme in South Africa
Source: PLoS One. 2010 Feb 17;5(2):e9183. doi: 10.1371/journal.pone.0009183 (PMC2822855; doi:10.1371/journal.pone.0009183)
Supplement: Table S1 — Associations between patient characteristics and hepatotoxicity. (0.06 MB DOC) [file pone.0009183.s001.doc]

| **Table S1: Associations Between Patient Characteristics and Hepatotoxicity** | | | | | | | |
| --- | --- | --- | --- | --- | --- | --- | --- |
|  |  |  |  |  |  |  |  |
|  | **Univariate** | | |  | **Multivariate** | | |
|  | HR | 95% CI | p |  | HR | 95% CI | p |
|  |  |  |  |  |  |  |  |
| Males | 1.1 | (0.54-3.4) | 0.854 |  | 1 | (0.4-3.8) | 0.756 |
|  |  |  |  |  |  |  |  |
| Age on starting NVP based ART, years | 1.0 | (1.0-1.1) | 0.606 |  | 1.0 | (1.0-1.1) | 0.540 |
|  |  |  |  |  |  |  |  |
| Baseline CD4 count (cell/µl) | 1.0 | (1.0-1.0) | 0.948 |  | 1.0 | (1.0-1.0) | 0.834 |
|  |  |  |  |  |  |  |  |
| 6 month CD4 count increase >100 cells/ul | 0.9 | (0.4-2.3) | 0.857 |  | 0.9 | (0.4-2.3) | 0.865 |
|  |  |  |  |  |  |  |  |
| Concurrent TB infection | 0.7 | (0.2-2.3) | 0.507 |  | 0.6 | (0.2-2.3) | 0.489 |
|  |  |  |  |  |  |  |  |
| PMTCT | 1.1 | (0.6-1.7) | 0.832 |  |  |  |  |
|  |  |  |  |  |  |  |  |
| Baseline weight ≤ 60kg | 1.1 | (0.4-3.0) | 0.786 |  |  |  |  |
|  |  |  |  |  |  |  |  |
| NVP, Nevirapine. ART, antiretroviral therapy. TB, tuberculosis. PMTCT, preventing mother-to-child transmission program. | | | |  |  |  |  |
|  |  |  |  |  |  |  |  |
