# Supplementary material for: Nevirapine-Associated Early Hepatotoxicity: Incidence, Risk Factors, and Associated Mortality in a Primary Care ART Programme in South Africa
Source: PLoS One. 2010 Feb 17;5(2):e9183. doi: 10.1371/journal.pone.0009183 (PMC2822855; doi:10.1371/journal.pone.0009183)
Supplement: Table S2 — Associations between patient characteristics and mortality. (0.06 MB DOC) [file pone.0009183.s002.doc]

| **Table S2: Associations Between Patient Characteristics and Mortality*** | | | | | | | |
| --- | --- | --- | --- | --- | --- | --- | --- |
|  | **Univariate** | | |  | **Multivariate** | | |
|  | HR | 95% CI | p |  | HR | 95% CI | p |
|  |  |  |  |  |  |  |  |
| Males | 1.1 | (0.6-2.0) | 0.693 |  | 1.3 | (0.7-2.3) | 0.412 |
|  |  |  |  |  |  |  |  |
| Age on starting NVP based ART, years | 1.0 | (1.0-1.0) | 0.490 |  | 1.0 | (1.0-1.0) | 0.345 |
|  |  |  |  |  |  |  |  |
| Baseline CD4 count >100 cells/ul | 0.4 | (0.2-0.6) | <0.001 |  | 0.4 | (0.2-0.6) | <0.001 |
|  |  |  |  |  |  |  |  |
| Concurrent TB infection | 0.9 | (0.5-1.6) | 0.807 |  |  |  |  |
|  |  |  |  |  |  |  |  |
| PMTCT | 0.8 | (0.6-1.1) | 0.184 |  |  |  |  |
|  |  |  |  |  |  |  |  |
| Hepatotoxicity | 2.6 | (0.9-9.3) | 0.098 |  | 2.5 | (0.-7.9) | 0.126 |
|  |  |  |  |  |  |  |  |
| Grade 3 or 4 ALT at baseline | 3.5 | (0.5-25.1) | 0.215 |  | 2.4 | (0.3-17.5) | 0.383 |
|  |  |  |  |  |  |  |  |
| NVP, Nevirapine. ART, antiretroviral therapy.WHO, World Health Organization. TB, tuberculosis. PMTCT, preventing mother-to-child transmission program. ALT, alanine aminotransferase | | | | | | | |
|  |  |  |  |  |  |  |  |
| *Analysis restricted to the first 6 months |  |  |  |  |  |  |  |
|  |  |  |  |  |  |  |  |
|  |  |  |  |  |  |  |  |
